# Feasibility and staff acceptability of implementing Xpert HIV-1 viral load point-of-care testing: a pilot study in San Francisco

**DOI:** 10.1186/s12879-024-10384-2

**Published:** 2025-01-06

**Authors:** Kelvin Moore Jr., Noelle Le Tourneau, Jasmin Alvarez, Santos Rodriguez, Janessa Broussard, Pierre-Cédric Crouch, Jorge Roman, Patricia Defechereux, Jason Bena, Kimberly A. Koester, Lissa Moran, Christopher Pilcher, Robert Grant, Katerina A. Christopoulos

**Affiliations:** 1 University of California, San Francisco, San Francisco, CA USA; 2 San Francisco AIDS Foundation, San Francisco, CA USA; 3 Gladstone Institutes, San Francisco, CA USA; 4 Division of HIV, Infectious Disease, and Global Medicine, Zuckerberg San Francisco General Hospital, University of California San Francisco, 995 Potrero Ave, Fourth Floor, San Francisco, CA 94110 USA

**Keywords:** HIV, Viral load, Point-of-care testing, Antiretroviral therapy, Acute HIV infection

## Abstract

**Background:**

Point-of-care HIV viral load testing may enhance patient care and improve HIV health services. We aimed to evaluate the feasibility and acceptability of implementing such testing in a high-volume community sexual health clinic in the United States.

**Methods:**

We conducted a cross-sectional, mixed-methods study. Remnant venipuncture specimens from clients undergoing HIV and other sexual health screenings were analyzed using the Xpert^®^ HIV-1 Viral Load assay. Results were compared to COBAS^®^ AmpliPrep/COBAS^®^ TaqMan^®^ HIV-1 Test. Clinical staff observations, study meeting notes, and two semi-structured in-depth interviews with clinical staff were used to understand perspectives on incorporating this testing into clinical practice.

**Results:**

We analyzed 113 samples from 111 clients. The Xpert assay showed excellent agreement with COBAS, with no clinically significant difference in viral load measurements. Clinical staff found Xpert testing acceptable, based on its ability to provide rapid, accurate test results and potential for bridging patient care gaps. Respondents noted that this testing would be particularly beneficial for individuals in whom barriers to care engagement may complicate follow-up. Challenges in implementation included machine errors as well as concerns related to staff workload, testing logistics, and the need for comprehensive equipment training.

**Conclusions:**

This study represents the first effort in the United States to describe the feasibility of HIV viral load point-of-care testing in routine care. While the Xpert demonstrated comparable results to standard-of-care testing and staff found it acceptable, further work is needed to develop the workflow and implementation strategies that would enable real-time use and improved patient care.

**Clinical trial:**

Not applicable.

**Supplementary Information:**

The online version contains supplementary material available at 10.1186/s12879-024-10384-2.

## Background

Point-of-care tests (POCTs)–defined as tests that are rapid, near-patient, and can dictate clinical management–have garnered considerable attention in the realm of HIV testing and treatment as they hold potential to circumvent barriers (e.g., central laboratory processing; extended turnaround time) surrounding confirmatory testing as well as provide real-time information about viral load (VL) status [1–4]. The World Health Organization has approved two quantitative HIV VL POCTs for use in resource-limited settings, the Xpert^®^ HIV-1 Viral Load (Xpert) and the m-PIMA™ HIV-1/2 Viral Load Test [5]. These assays have been used for VL monitoring in low- and middle-income countries proving helpful in allowing clinicians to rapidly assess viremia in order to guide antiretroviral (ART) adherence counseling [6–10].

While current guidelines from the Centers for Disease Control and Prevention and Association of Public Health Laboratories state that laboratories should perform an HIV-1/HIV-2 antibody differentiation supplemental immunoassay after reactive antigen/antibody screening immunoassays [11], HIV VL POC testing may shorten confirmation time for individuals newly diagnosed with HIV or identify acute HIV in those with discordant test results. Such testing may also prove beneficial when considering ART management for individuals with known HIV whose VL values are unknown, and especially those whereby structural circumstances preclude routine care engagement. Moreover, this testing may be of use for long-acting injectable cabotegravir pre-exposure prophylaxis (PrEP) initiation and monitoring [12–14].

As part of a study evaluating the impact of rapid ART [15] on linkage to care and viral suppression in a high-volume community HIV testing site, we sought to assess feasibility and staff acceptability of deploying an HIV VL POCT and assess assay performance vis-à-vis diagnostic standard-of-care (SOC) testing.

## Methods

### Study design and setting

This cross-sectional, pilot study took place at Magnet––a sexual health and wellness clinic in San Francisco, California. Magnet provides drop-in and scheduled services at no cost to approximately 8,500 clients, mostly men who have sex with men. Services include sexually transmitted infection (STI) testing and treatment, HIV testing and linkage to care, pre-exposure prophylaxis (PrEP)/post-exposure prophylaxis (PEP) services, gender-affirming care, anal health services, and hepatitis C testing and treatment. Additionally, Magnet initiates rapid ART for individuals newly diagnosed with HIV [15] and provides reengagement ART services for individuals with a lapse or threatened lapse in HIV care [16].

### Standard-of-care testing protocol

Magnet uses the FDA-approved HIV 1/2 STAT-PAK^**®**^ (STAT-PAK, Chembio Diagnostics, Inc., Medford, NY) and the Geenius^**®**^ HIV 1/2 supplemental assay (Geenius, Bio-Rad, Hercules, California) for confirmation of a positive STAT-PAK result. Clients deemed to have high risk for acute HIV infection undergo pooled HIV RNA testing at the San Francisco Department of Public Health laboratory [17, 18]. Quantitative HIV RNA testing is performed using the COBAS^®^ AmpliPrep/COBAS^®^ TaqMan^®^ HIV-1 Test, Version 2.0 (COBAS). Results of pooled and quantitative HIV RNA testing are available in 3–4 days.

### HIV point-of-care viral load testing

The Xpert^®^ HIV-1 Viral Load (Xpert, Cepheid, Sunnyvale, California) assay has a linear quantitation range of 40 copies/mL to 10,000,000 copies/mL. It requires a minimum of 1 mL of plasma and has an approximate run time of 91 minutes [19]. We used two GeneXpert^®^ IV devices which collectively have the operational throughput of 42 samples per 8-hour shift [20]. We employed off-label use to assess Xpert’s utility as a confirmatory diagnostic tool [19]. While the Xpert package insert refers to ‘near-patient’ testing, we use the term ‘point-of-care’ in this paper, aligning with the CLIA definition that emphasizes the location of testing at the site of patient care, irrespective of test complexity and waiver status [21, 22].

### Data collection

Remnant specimen from clients undergoing HIV/STI testing or PrEP/PEP/HIV care was used from whole blood samples collected in lavender BD Vacutainer EDTA tubes and centrifuged at 3500 RPM for 10 min to isolate plasma supernatant. Using Xpert pre-packaged pipettes, a research coordinator aliquoted 1.2 mL of the supernatant either directly into the Xpert cartridge for analysis or into 2 mL microcentrifuge test tubes for storage.

Samples were stored in a designated refrigerator at 4 °C. Results of testing were neither disclosed to clients nor used to guide clinical care, therefore samples were batched in groups of four at the end of each clinic day. If samples could not be analyzed on the same day as collection, they were stored at -1 °C and thawed for 30 min before preparing the Xpert cartridge.

HIV RNA results were exported from the Xpert to an Excel file. Demographic information and results of other HIV testing performed on the same day were extracted from the electronic medical record (EMR).

### Data analysis

**Fig. 1:**
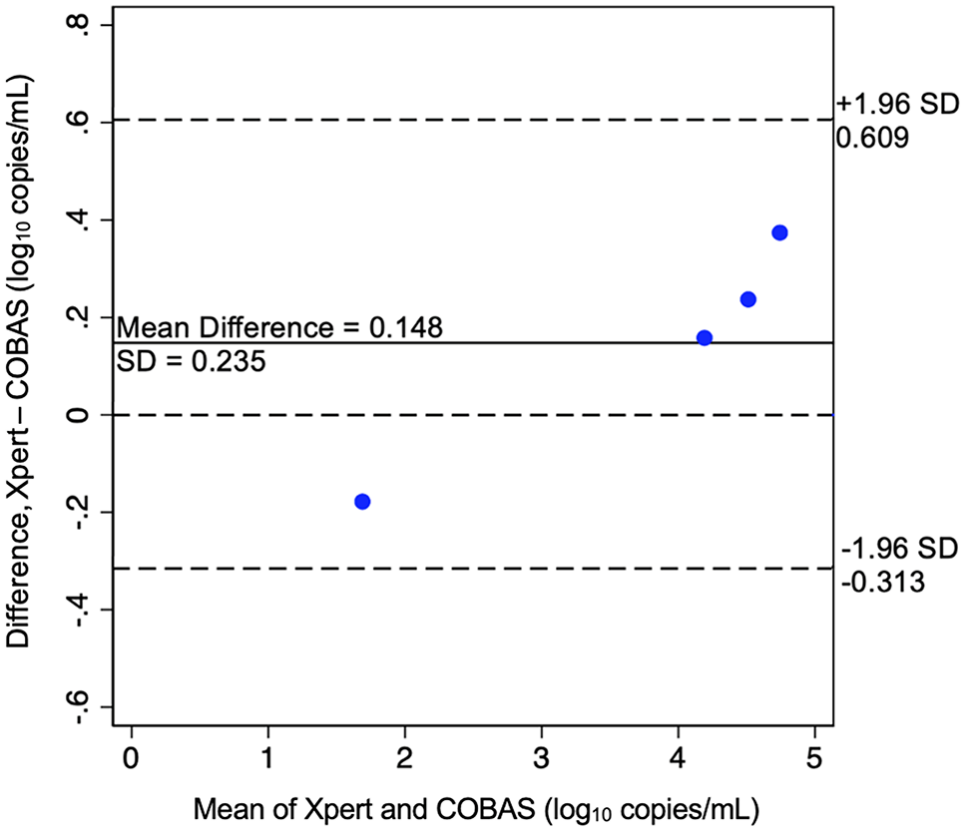
Bland-Altman Plot Concordance between Xpert and COBAS assays

We used descriptive statistics to summarize demographic characteristics of clients. Frequency tables were generated for the number of samples collected, the number of tests conducted, the number of errors produced, and the number of detectable RNA results. All laboratory test results for an individual with detectable HIV RNA on the Xpert software were reviewed and summarized (Table 1). A Bland-Altman plot compared VL measurements (using log_10_ copies/mL) between Xpert and COBAS (Fig. 1) for assay validation. Upper and lower limits of agreement were defined as ± 1.96 standard deviations of the mean VL difference between the two assays [23]. Data were managed with Stata statistical software (Stata 16.1).

**Table 1:** Results of Standard of Care and Xpert HIV-1 testing

Sample ID	Testing Category	HIV Antibody (STAT-PAK)	Geenius HIV ½ Supplemental Assay	Xpert HIV-1 Viral Load (copies/mL)	Xpert HIV-1 Viral Load (log_10_ copies/mL)	COBAS HIV-1 Viral Load (copies/mL)	COBAS HIV-1 Viral Load (log_10_ copies/mL)
1	Known diagnosis-Interim ART	Reactive	N/A	< 40	1.6	60	1.778
2	Sexual Health Screen	Reactive	HIV-1 Positive	18,600	4.27	12,930	4.112
3	New Diagnosis	Reactive	HIV-1 Positive	42,400	4.63	24,730	4.393
4	New Diagnosis	Reactive	HIV-1 Positive	84,900	4.93	36,010	4.556
5	Known diagnosis- Interim ART	Reactive	N/A	337,000	5.53	Did not want labs to be sent	Did not want labs to be sent

### Qualitative evaluation

The qualitative evaluation utilized several methods including clinical staff observations, document review (meeting notes/minutes), and two semi-structured in-depth interviews: one with the research coordinator responsible for deploying the Xpert assay to assess the experience of using the test and another with a clinic nurse practitioner to understand perspectives on incorporating this technology into clinical practice. The interview guide (Supplementary File) utilized in this study was specifically developed for the purposes of this study and has not been previously published elsewhere. Professional qualitative researchers not known to Magnet staff conducted the interviews. Interviews were audio-recorded and transcribed with accompanying field notes to capture salient observations. The first and last author read the qualitative materials and discussed findings to generate an analytic memo, using study meeting minutes to supplement emerging findings.

### Ethical approval

The University of California, San Francisco Institutional Review Board approved this study. The study protocol was reviewed and approved under CHR# 18-25758. As this study utilized remnant specimen from venipuncture conducted in the course of clinical care and results were not used for clinical management, the UCSF IRB granted a waiver of informed consent for Magnet clients. Staff provided informed consent to participate in interviews outside of work hours and were compensated $50.

## Results

### Participant characteristics

Between July 2021 – October 2021, Xpert testing was performed on 113 samples from 111 clients. Clients from whom samples were collected were representative of the population regularly seen at Magnet. Namely, they were predominantly male (86.5%), non-white (55.8%), ‘gay/lesbian/queer’ (89.2%), in stable housing (90.1%), based in San Francisco (65.8%), and insured (55.3%). The median age was 32 (IQR 27–38). The majority of samples (45%) were from PrEP initiation visits followed by sexual health screenings (32%) whereby individuals underwent full STI panel testing with or without a nurse visit. A smaller proportion of samples were from post-exposure prophylaxis (PEP) initiation visits (8%), clients exhibiting symptoms of acute HIV infection and recent exposure (5%), clients restarting ART (3%), and clients newly diagnosed with HIV (2%).

Eight samples resulted in system errors on the Xpert software––namely, five assay-specific termination errors due to inadequate sample volume, two probe check failures, and one syringe drive motion error. The median total run-time for non-error samples was 89.9 min. Median total run-time for samples that resulted in errors was 41.3 min.

HIV-1 RNA was detected in five samples, one of which yielded < 40 copies/mL (Table 1). For those who had detectable HIV RNA on Xpert testing, reasons for SOC testing were: interim ART refills for individuals with known diagnoses of HIV (*N* = 2), individuals with newly diagnosed HIV (*N* = 2), and routine sexual health screening (*N* = 1). One client with previously diagnosed HIV did not wish to have further SOC testing performed after the reactive Statpak result.

The Xpert assay showed excellent agreement with COBAS (mean log_10_ copies/mL difference of 0.148 ± 0.235) as demonstrated in Fig. 1.

### Benefits and challenges of point-of-care testing

In the qualitative evaluation, the main benefit identified was faster turnaround time for results and the theoretical facilitation of immediate care and treatment. POC testing would aid in diagnosis, especially in cases of acute HIV infection, and provide important and timely information for clients to assess their VL after ART initiation. For clients with known HIV, POC testing could inform the initiation or adjustment of same-day ART. Being able to discuss the clinical implication of a high VL in real time was seen as a plus, with the potential to enhance client understanding of their clinical status. One respondent highlighted how VL POCTs enhance HIV counseling by tailoring the information provided to clients, thereby encouraging adherence:

For someone coming in for a one-month follow-up, seeing their viral load drop—or even become undetectable—can be really encouraging. Sometimes it takes a while to connect to care… Usually, it’s more like six weeks, but adherence counseling and encouragement can be useful there. And, on the flipside, if you’re thinking about community spread and reducing, you know, community viral load, that’s important information for someone to know too—that they may be infectious still.

Another benefit was decreased external reliance on centralized laboratories, with reductions in associated laboratory costs and decreased risk of specimen mishandling. One respondent highlighted the utility and impact of in-house processing of VL:

If we had this today at Magnet Clinic — a fully fleshed-out, community-supported clinic — and could implement it along with our lab process, we wouldn’t have to send out every single sample for confirmatory testing to third parties… We would have primary, secondary, and tertiary-level data right there on-site, improving contact tracing and rapid [ART] start.

Challenges identified were related to staff workload and testing logistics. Workflow development would need to account for timing of testing, results notification, and space for the machine. Comprehensive equipment training was highlighted, with specific counseling to avoid bubble aspiration when aliquoting the plasma supernatant. Respondents also noted the requirement for stable electrical connectivity, which could complicate use in clinical settings where power is not consistent. Finally, there was the concern that clients may not want to wait 90 min for results:

The 90 min is a real – it seems like a barrier to me. Because, generally, nobody’s here for 90 min… More often, they’re in – out of here within 60–70 min, at least from the time they get their blood draw.

In discussing next steps for Xpert testing, respondents indicated that integrating several HIV/STI assays into one cartridge could optimize testing efficiency and cost-effectiveness.

## Conclusions

In this pilot study assessing feasibility and staff acceptability of implementing Xpert POC VL testing at a high-volume U.S. sexual health clinic, we demonstrated that such testing could be feasibly integrated into SOC workflows. Our findings suggest that other sites in the U.S. may benefit from exploring similar integration of testing into HIV prevention and treatment activities. Given that SOC HIV RNA testing typically takes 3–4 days to return results [24], implementing POC VL testing could reduce this turnaround time and potentially improve patient care.

Generally, the Xpert results were similar to those of the COBAS assay and there was no clinically relevant difference in VL measurements, defined as < 0.5 log_10_ copies/mL [25], for all samples. These findings, based on a limited sample size, require validation with larger studies to confirm the agreement between the Xpert and COBAS platforms. We did not validate diagnostic performance given our sample size. While detection of acute HIV infection through use of pooled HIV RNA testing is not uncommon at Magnet, we found no discordant (STAT-PAK negative/Xpert reactive) cases, albeit in a small sample. Xpert did detect a sample of < 40 copies/mL in Sample 1 while the COBAS assay reported a VL of 60 copies/mL, which would likely prompt adherence assessment and repeat VL testing at a later date.

Our qualitative review yielded several important insights. First, the data highlighted several potential benefits identified by staff regarding rapid and accurate HIV VL POC testing: facilitation of timely linkage to care, potential reduction of HIV transmission, and mitigation of gaps in the HIV testing and treatment cascade due to lack of patient follow-up. Moreover, HIV VL POC testing could enable initiation of effective ART in real time by identifying cases of ART failure and acute HIV.

We found the need to attend to logistical and access considerations. Notably, careful staff training on equipment operation is key to ensure accurate and effective Xpert use. Additional lab space is needed for the Xpert machinery which could pose a problem in clinical settings where there may be space constraints. We found a potential need for standardized pipetting equipment and, while uncommon, system errors may require additional sample. Strategies to encourage clients to wait upwards of 90 min for a result may be necessary. Remote results notification via phone, text, or mobile application could be an option, but would not work for individuals without consistent phone and/or internet access. Finally, the need for an uninterrupted power source could pose a challenge in areas with inconsistent electrical infrastructure.

Another key consideration is cartridge cost. Integrating HIV testing with STI testing may reduce cartridge costs and optimize testing efficiency. Multiplex STI POCTs are currently being explored and developed [26]. Additionally, one must consider the regulatory hurdles related to CLIA certification such as testing complexity categorization and routine quality assurance inspections [27]. While necessary for appropriate, accurate, and safe testing, under- resourced communities may not have the clinical infrastructure to support such certification. As the FDA recently permitted the first POC Hepatitis C RNA Test, this approval may help pave the way for granting similar permissions for emerging HIV POCTs [28]. Lastly, there is need to assess costs of deploying POC VL testing and whether such approaches may be cost-effective, particularly in under-resourced settings.

In summary, our study represents the first U.S. effort to understand the feasibility and staff acceptability of implementing Xpert HIV VL POCTs at a community HIV testing site, adding to existing Xpert literature [29–37]. Other sites in the U.S. are similarly interested in exploring Xpert integration into HIV prevention and treatment activities [38]. Further work is needed in U.S. clinical settings to develop workflows that account for key implementation factors (e.g., staff resources, EMR documentation, client acceptance of wait time, laboratory certifications, and cost considerations) that would allow for real-time use and improved patient care [39]. From a client acceptability standpoint, clear communication about the benefits of rapid VL results and potential wait times during visits is crucial. It is also critical to evaluate client acceptability of such testing. Incorporating Xpert into routine workflows may require adjustments to patient flow to minimize delays, such as offering the test at the beginning of a visit to allow results to be processed by the end. Additionally, enhancing client-staff interactions by training staff to provide real-time adherence counseling or linkage to care based on rapid VL results can maximize the clinical utility of this approach.

## Electronic supplementary material

Below is the link to the electronic supplementary material.

Supplementary File “Feasibility of VL Testing at Magnet Point of Care VL Testing Interview Guide”. The interview guide utilized in this study was specifically designed to explore the feasibility and acceptability of point-of-care viral load testing in a U.S. clinical setting. The guide includes questions aimed at understanding healthcare providers’ experiences, perspectives, and logistical considerations surrounding such testing. This tool has not been previously published and is provided as a supplementary file to aid in replicability and transparency.

## Data Availability

The dataset generated and analyzed during the study are not publicly available due to the small sample size and the sensitive nature of the information collected, which could potentially compromise participant privacy and confidentiality. However, the data may be available from the corresponding author upon reasonable request, subject to appropriate data sharing agreements and ethical considerations.

## Abbreviations

ART: Antiretroviral Therapy
COBAS: COBAS^®^ AmpliPrep/COBAS^®^ TaqMan^®^ HIV-1 Test, Version 2.0
EMR: Electronic Medical Record
Geenius: Geenius^®^ HIV 1/2 supplemental assay
HIV: Human Immunodeficiency Virus
PEP: Post-exposure Prophylaxis
POC: Point-of-Care
POCTs: Point-of-Care Tests
PrEP: Pre-exposure Prophylaxis
SOC: Standard-of-Care
STAT-PAK: HIV 1/2 STAT-PAK^®^
STIs: Sexually Transmitted Infections
VL: Viral Load
Xpert: Xpert^®^ HIV-1 Viral Load
