# Supplementary material for: Feasibility and staff acceptability of implementing Xpert HIV-1 viral load point-of-care testing: a pilot study in San Francisco
Source: BMC Infect Dis. 2025 Jan 6;25:26. doi: 10.1186/s12879-024-10384-2 (PMC11702105; doi:10.1186/s12879-024-10384-2)
Supplement: Supplementary file 1 — Supplementary File “Feasibility of VL Testing at Magnet Point of Care VL Testing Interview Guide”. The interview guide utilized in this study was specifically designed to explore the feasibility and acceptability of point-of-care viral load testing in a U.S. clinical setting. The guide includes questions aimed at understanding healthcare providers’ experiences, perspectives, and logistical considerations surrounding such testing. This tool has not been previously published and is provided as a supplementary file to aid in replicability and transparency. [file 12879_2024_10384_MOESM1_ESM.pdf]

Feasibility of VL Testing at Magnet
Point of Care VL Testing
Interview Guide

This interview will help me to understand your perspective on the idea of using a viral load testing machine in clinical practice, its pros and cons, and I'd also like to find out what kind of support you might need to optimize its usefulness.

There is no direct benefit to you for participating, though you will be provided a small incentive as a token of appreciation for your time. You can stop the interview at any time, without penalty. We will be recording the interview so that your responses can be transcribed. Your identity will

Defining the Tool This point of care (or POC) viral load testing is a new test that allows detection and quantification of blood plasma viral RNA (the viral load) in around 90 minutes right here in the clinic, without a need to send out specimens. The test uses a strict minimum of 1.2 mL plasma which is spun for 10 min from an EDTA tube within 8 hours of collection. Once collected, the plasma can be stored up to 24hrs at 4C or for longer periods at -20C. Specimens can be run in batches at the end of the day however once the plasma specimens are loaded on the test cartridge, the test must be run within 1 hr. This rapid viral load test is designed to detect the viral RNA on an automated system using what is called real time reverse transcription PCR (RT- PCR) and requires minimal infrastructure and training. The system integrates all the steps of this RT-PCR approach in a compact instrument and provides results immediately which may help with immediate and on-site confirmation of viral infection. Though these tests have been widely used around the world, they are not yet approved by the FDA for clinical care.

First let me start by asking you some basics:

1) What **do you think about the use of** point of care viral load testing in general?
2) How long have you had the [VL machine] at SFAF?
3) Have you had any direct interaction with the [VL machine] and if so, can you describe what that's looked like?
4) What was your initial impression of the [Cepheid machine] when you first heard that Strut would be receiving one?
5) How has that impression shifted now that it has been onsite for a while?
6) Have do you see yourself using this test in your practice here at SFAF?
7) What types of patients would you use [POC VL] testing with?
    a) What makes those patients good candidates for [POC VL] testing?
    b) Describe situations where you might hesitate using [POC VL] testing.
8) How would you handle the logistics of having a 90-minute processing time window? Probe: quality controls issues/protocol, space issues, operability
9) What changes to the infrastructure would be necessary to optimize the [use of POC VL] testing?

10) What concerns do you have about the use of this technology?

11) To what extent do you think the providers here at SFAF endorse the use of POC VL testing? What makes you say that?

12) Who benefits from POC VL testing?

    a) What are the benefits of treating someone during the acute phase?

    b) Do all staff know about these implications?

13) What information would you like to have about [POC VL] testing that you don't currently have?

    a) How would you like to receive more info –website, webinar, articles, peers, other?

14) How important is it that a testing site has access to this technology?

15) What advice do you have for the implementation of [POC VL] testing in other locations?

16) Is there anything else you think I should know about how you feel about using [POC VL] testing at SFAF?
